# Bergenin inhibits growth of human cervical cancer cells by decreasing Galectin-3 and MMP-9 expression

**DOI:** 10.1038/s41598-024-64781-3

**Published:** 2024-07-03

**Authors:** Ravi Chauhan, Lakshay Malhotra, Ashna Gupta, Gunjan Dagar, Mohini Mendiratta, Tariq Masoodi, Sheema Hashem, Sara Al Marzooqi, Dayasagar Das, Shahab Uddin, Abdul Samath Ethayathulla, Muzafar A. Macha, Ammira Al-Shabeeb Akil, Ranjit Kumar Sahoo, Ekta Rai, Ajaz A. Bhat, Mayank Singh

**Affiliations:** 1 Department of Medical Oncology (Lab.), Dr. BRAIRCH, All India Institute of Medical Sciences (AIIMS), New Delhi, 110029 India; 2 Department of Medical Oncology, Dr. BRAIRCH, All India Institute of Medical Sciences (AIIMS), New Delhi, India; 3 Department of Biophysics, All India Institute of Medical Sciences, New Delhi, India; 4 Department of Biochemistry, Sri Venkateswara College, University of Delhi, New Delhi, India; 5 Laboratory of Cancer Immunology and Genetics, Sidra Medicine, Doha, Qatar; 6 Department of Human Genetics, Sidra Medicine, Doha, Qatar; 7 Department of Human Genetics-Precision Medicine in Diabetes, Obesity and Cancer Program, Sidra Medicine, Doha, Qatar; 8 Department of Medicine, NYU Langone Health, New York, 10016 USA; 9 Translational Research Institute, Academic Health System, Hamad Medical Corporation, Doha, Qatar; 10 Watson-Crick Centre for Molecular Medicine, Islamic University of Science and Technology, Pulwama, Jammu and Kashmir India; 11 School of Life Sciences Jawahar Lal Nehru University, New Delhi, India

**Keywords:** Bergenin, Cervical cancer, HPV, Galectin 3, Matrix metallo protease 9, Cancer, Molecular medicine

## Abstract

Cervical cancer is still the leading cause of cancer mortality worldwide even after introduction of vaccine against Human papillomavirus (HPV), due to low vaccine coverage, especially in the developing world. Cervical cancer is primarily treated by Chemo/Radiotherapy, depending on the disease stage, with Carboplatin/Cisplatin-based drug regime. These drugs being non-specific, target rapidly dividing cells, including normal cells, so safer options are needed for lower off-target toxicity. Natural products offer an attractive option compared to synthetic drugs due to their well-established safety profile and capacity to target multiple oncogenic hallmarks of cancer like inflammation, angiogenesis, etc. In the current study, we investigated the effect of Bergenin (C-glycoside of 4-*O*-methylgallic acid), a natural polyphenol compound that is isolated from medicinal plants such as *Bergenia crassifolia*, *Caesalpinia digyna,* and *Flueggea leucopyrus*. Bergenin has been shown to have anti-inflammatory, anti-ulcerogenic, and wound healing properties but its anticancer potential has been realized only recently. We performed a proteomic analysis of cervical carcinoma cells treated with bergenin and found it to influence multiple hallmarks of cancers, including apoptosis, angiogenesis, and tumor suppressor proteins. It was also involved in many different cellular processes unrelated to cancer, as shown by our proteomic analysis. Further analysis showed bergenin to be a potent-angiogenic agent by reducing key angiogenic proteins like Galectin 3 and MMP-9 (Matrix Metalloprotease 9) in cervical carcinoma cells. Further understanding of this interaction was carried out using molecular docking analysis, which indicated MMP-9 has more affinity for bergenin as compared to Galectin-3. Cumulatively, our data provide novel insight into the anti-angiogenic mechanism of bergenin in cervical carcinoma cells by modulation of multiple angiogenic proteins like Galectin-3 and MMP-9 which warrant its further development as an anticancer agent in cervical cancer.

## Introduction

Cervical cancer poses a significant threat to the health of women and is leading cause of mortality in womens worldwide. Cervical cancer is primarily associated with high-risk human papillomavirus (HPV) infection, among other risk factors^1^. According to the Globacon 2020 cancer facts, 6,04,127 new cervical cancer cases and 3,41,831 deaths were reported among all age groups worldwide. Furthermore, cervical cancer is the fourth most common malignancy among females, with a 13.3% incidence rate and a 7.3% mortality rate. The risk factors besides infection with HPV for cervical cancer include early sexual maturity, polygamy, early pregnancy, multiple pregnancies, smoking, and immunosuppression^2^*.* Pre-cancerous changes associated with cervical cancer can be detected by the pap smear test and is recommended for preventive screening of cervical cancer worldwide. High-risk Human Papillomavirus persistent infection and its associated oncogenesis by inactivation of tumor suppressors p53 and Rb has been established as a major pathway for tumor development., Despite significant advances in our understanding of cervical cancer as a potentially preventable disease, major improvements in cervical cancer therapeutics have to be achieved. Thus, the disease burden remains high, particularly in developing countries where vaccine coverage is significantly low^3^. The treatment strategy for cervical cancer is determined by the stage and extent of cervical cancer progression, which generally includes a combination of surgery, radiation, and chemotherapy. Various therapeutic modalities have been validated in cervical cancer, including therapeutic agents against different molecules regulating different biological pathways i.e., cell cycle progression, cell growth, DNA repair, and angiogenesis^4^. Tumor growth and progression depends on angiogenesis which is the formation of new blood vessels and involves the migration, growth, and differentiation of endothelial cells, and is considered one of the essential hallmarks of cancer^5^. Targeting angiogenesis has been considered one of the valid strategies to combat cancer and drugs have been developed against angiogenic factors like VEGF (Vascular endothelial growth factors). Drugs like bevacizumab and pazopanib have received approval for the treatment of gynecological malignancies and a number of other drugs are currently being tested in trials targeting angiogeneic pathways in cervical cancer^4,6^*.*

Different Studies have revealed that various plant extracts possess a repertoire of anti-cancer properties with potential prospect of application in modern chemotherapeutics as a radio and chemosensitizers^7,8^. Drug resistance, recurrence, and metastasis have been associated with new-age cancer treatments like monoclonal antibodies because of inherent plasticity of cancer cells and selection pressure. Additionally conventional chemotherapy backbone is based on Carboplatin/Cisplatin, which targets the rapidly dividing cells, including normal cells resulting in significant off-target toxicities. Synthetic drug-based toxicity needs to be addressed, and safer drugs with high efficacy and low side effects to treat cervical cancer need to be validated. Plant-derived natural products have traditionally been used to treat various human conditions. They have been evaluated in the last two decades as potential anticancer drugs that preferentially kill tumor cells without causing severe side effects^9^. A growing number of studies have shown that natural products can have possible anti-cervical-cancer effects via various mechanisms, including tumor-cell proliferation inhibition, apoptosis induction, angiogenesis and telomerase activity suppression, immunity enhancement, and multi-drug resistance reversal. It been shown that many natural products like Curcumin, Berberine, Allicin, Withaferin, etc., target cervical cancer cells by inhibiting multiple oncogenic pathways^10–12^.

**Figure 1:**
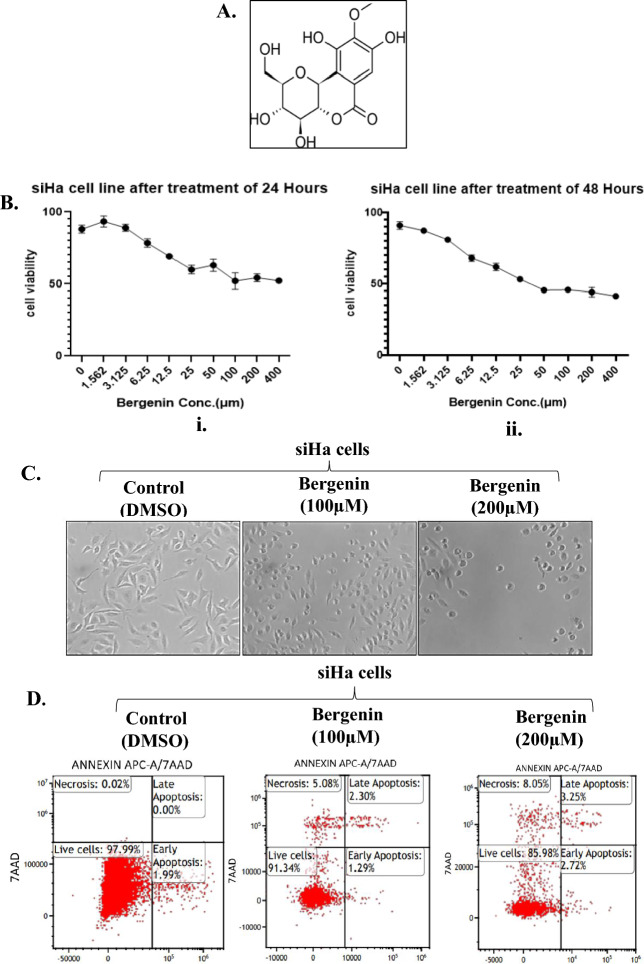
Bergenin reduces viability in cervical carcinoma cells (**A**) Chemical structure of Bergenin. (**B**) Cell viability was assessed in SiHa cells after treatment with varying concentrations of bergenin for (i) 24 h and (ii) 48 h through MTT assay. (**C)** Morphological changes in SiHa cells after treatment with bergenin (100 µM and 200 µM) for 24 h. (**D)** Representative dot plots of Annexin-V/7AAD of SiHa cells before and after bergenin(100 µM and 200 µM) treatment for 48 h.

Bergenin, a natural secondary metabolite also named as cuscutin (Fig. 1A), is isolated from different parts of several plants and is one of the active ingredients in herbal and ayurvedic formulations. It exhibits anti-viral, anti-fungal, anti-tussive, anti-plasmodial, anti-inflammatory, anti-hepatotoxic, anti-arrhythmic, anti-tumor, anti-ulcerogenic, anti-diabetic, and wound healing properties. Several bergenin derivatives have been isolated and/or synthesized and were found to possess a broad spectrum of pharmacological activities^13^. A recent study suggested that bergenin targets bladder cancer progression by targeting PPARγ/PTEN/Akt signal pathway^14^. Additionally, another study has provided evidence for theanticancer activity of the mangrove *Excoecaria agallocha* L. leaf extracts on human cervical cancer cell line (SiHa). Bergenin was found to be the major bioactive component identified in the leaf extracts, which inhibited the proliferation of SiHa cervical cancer cells through induction of autophagy and apoptosis in a concerted manner, with simultaneous stimulation of mitophagy and G2/M phase cell cycle arrest^15^. Another study using reverse docking found that Galectin-3 is a potential target of bergenin that plays a significant role in cell–cell adhesion, cell–matrix interactions, macrophage activation, angiogenesis, metastasis, and apoptosis in cancer^16^. In the current study, we were interested in identifying cellular targets that might be modulated by bergenin in cervical carcinoma cells. Since the majority of cervical cancers are associated with infection from high-risk human papilloma viruses so we carried out whole cell proteomics to understand different pathways modulated by bergenin in cervical cancer cells. Our analysis revealed multiple signaling pathways regulated by bergenin which were further validated by using biochemical assays, molecular docking and molecular dynamics simulation studies.

## Results

### Bergenin reduces cell viability in cervical cancer cells

Bergenin (Fig. 1A) has a wide spectrum of activity such as hepatoprotective, anti-inflammatory, immunomodulatory, antitumor, antiviral, antifungal properties while other studies have suggested antiproliferative and antitumor properties of bergenin^14^. A recent study has suggested that *Excoecaria agallocha* L. leaf extract have anticancer properties because of abundance of bergenin and it inhibits the growth and proliferation of cervical cancer cells^15^. So, we first reasoned what will be the cytotoxic effect of bergenin on HPV positive and negative cervical carcinoma cell lines. Therefore, using the MTT assay, we evaluated the effect of bergenin on the viability of SiHa which are HPV 16 positive, and C33A, which are HPV negative cervical cancer cells. SiHa and C33A cells were treated with bergenin for 24 and 48 h at different concentrations (0, 1.562, 3.12, 6.25, 12.5, 25, 50, 100, 200, and 400 µM). The results suggested that the viability of SiHa (Fig. 1B, Supplementary Fig. S1A) and C33A (Supplementary Fig. S2A). cells decreases in a dose-dependent manner. The results also indicate that the half maximum inhibitory concentration (IC50) of bergenin in both cervical cancer cells was around 100 μM. In our subsequent experiments, SiHa and C33A cells were treated with 100 µM and 200 μM of bergenin for 24 and 48 h. Besides reduced cell viability, Bergenin was also found to induce morphological alterations including cell shrinkage, fragmentation into membrane-bound apoptotic particles, and fast phagocytosis in SiHa (Fig. 1C and Supplementary Fig. S1B and C33a (Supplementary Fig. S2B) cells demonstrating the growth inhibitory potential of bergenin. Furthermore, we assessed whether the observed morphological alterations in cells treated with bergenin could be indicative of apoptotic cell death and sought to further investigate the impact of bergenin on cervical cancer cells. Additionally, we conducted annexin-PI staining to evaluate whether bergenin is inducing apoptosis in cervical cancer cells. In Fig. 1D, upon treatment in siHa cells with 100 µM and 200 µM of bergenin for 48 h, we observed indications of early apoptosis, late apoptosis, and cell necrosis using annexin-V-PE/7-AAD double staining. However, it's important to note that the percentage of cells in early and late apoptosis was relatively low, suggesting that significant apoptosis was not prominently observed. Specifically, following treatment with 100 µM of bergenin, the percentage of cells in early apoptosis was 1.29%, and in late apoptosis, it was 2.30%. After treatment with 200 µM of bergenin, the percentage of cells in early apoptosis increased to 2.72%, and late apoptosis reached 3.25%. In comparison, the control group showed a high percentage of live cells at approximately 97.99%, whereas after treatment with 100 µM of bergenin, approximately 91.34% of cells remained viable, and after 200 µM treatment, around 85.98% of cells remained viable. In summary, bergenin exhibited cytotoxic effects on both HPV-positive and HPV-negative cervical carcinoma cell lines, inducing morphological changes might be associated with apoptosis and cell death. This suggests the potential of bergenin as a candidate for further research and development in the context of cervical cancer treatment.

### Bergenin affects a distinct set of proteins in cervical carcinoma cells

**Figure 2:**
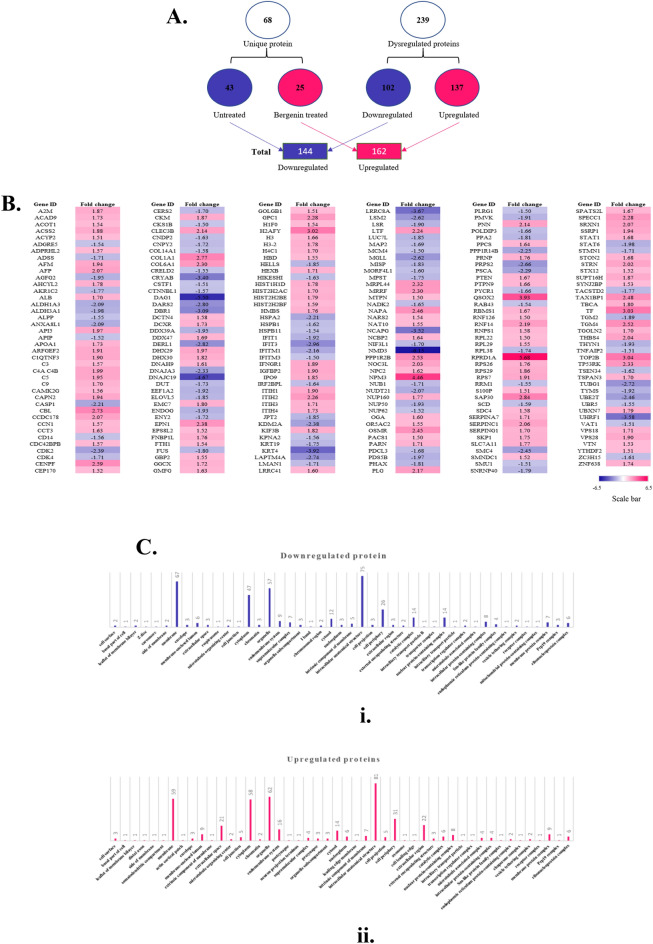
Bergenin causes upregulation and downregulation of distinct set of proteins in cervical cancer cells. (**A**) Pictorial representation of dysregulated proteins after Label-free quantification (LFQ) of the SiHa cells before and after 100 μM of bergenin treatment for 48 h. (**B**) The heat map shows the dysregulated proteins with ≥  ± 1.5-fold change. The pink color represents the upregulation, whereas the blue represents the downregulation. (**C**) Graphical representation of the cellular compartmentalization of the dysregulated proteins (i) Downregulated. (ii) Upregulated.

In order to convincingly understand the molecular pathway affected by Bergenin we evaluated the cellular proteome affected by bergenin, SiHa cervical carcinoma cells were treated with bergenin at a concentration of 100 μM for 48 h and processed for label-free protein quantification (LFQ) of the cellular proteome. The data obtained was analyzed for the dysregulation of different protein sets as compared to untreated cells. The LFQ analysis showed a total of 239 dysregulated proteins out of which 102 were downregulated and 137 were upregulated after the treatment of bergenin (Fig. 2A) (Supplementary Table S1). In addition, 68 different proteins between the treated and untreated samples were found. Comparison analysis showed that untreated cells had 43 distinct proteins, while one that had been treated had 25 distinct proteins. Additionally, the heat map analysis of the proteome indicated that many proteins involved in a different pathways are affected by bergenin (Fig. 2B). In our analysis, we found that proteins involved in inflammation, interleukins, JAK-STAT pathway (STAT6, COL14A1) are downregulated in bergenin-treated cells underlining its anti-inflammatory nature. Furthermore, we found that HSPA 2 (Heat shock protein family A member 2), a prominent cancer chaperonic protein, was downregulated in bergenin-treated cells. We also found downregulation of many proteins involved in proliferative pathways like p38 MAPK (HSPB1), EGFR (PRKC1, STAT6) and Histone H1 replication (EPB411.2) in bergenin-treated cells. These results suggest the anti-inflammatory and anti-proliferative potential of bergenin.

The heatmap of bergenin-treated cells also showed the upregulation of several proteins involved in diverse cellualr pathways (Fig. 2B) which includes PTEN (Phosphatase and tensin homolog), CDKN1A (Cyclin dependent kinase inhibitor 1A), and SMAD4 (SMAD Family member 4). All these have been shown to suppress tumor growth through different mechanisms^17^. We also found upregulation of CDKN1A (Cyclin dependent kinase inhibitor 1A) which is another protein that has an important role in cell cycle arrest^18^. We also found upregulation of SMAD4 (SMAD Family member 4) which comes in TGF beta signalling pathway and its deletion has been known to promote tumorigenesis^19^ We also found upregulation of STAT 1 in our dataset, which has been associated with variable outcomes in gynaecological cancers^20^. In addition to these, we found many other proteins like STX12, TATA-binding protein L1, and TOP 2B, which have a variable role in cancer. Overall, our analysis indicates that bergenin targets multiple cell signaling pathways in cervical carcinoma cells cumulatively leading to the inhibition of growth and induction of apoptosis. We were also interested in checking if bergenin also affects the spatial location of proteins. It was found that distinct sets of proteins located in the membrane, cytoplasm, organelles and anatomical structure were either upregulated or downregulated (Fig. 2Ci,ii) and these compartments have highest number of proteins targeted by bergenin.

### Bergenin affects different cell signaling pathways in cervical cancer cells

**Figure 3:**
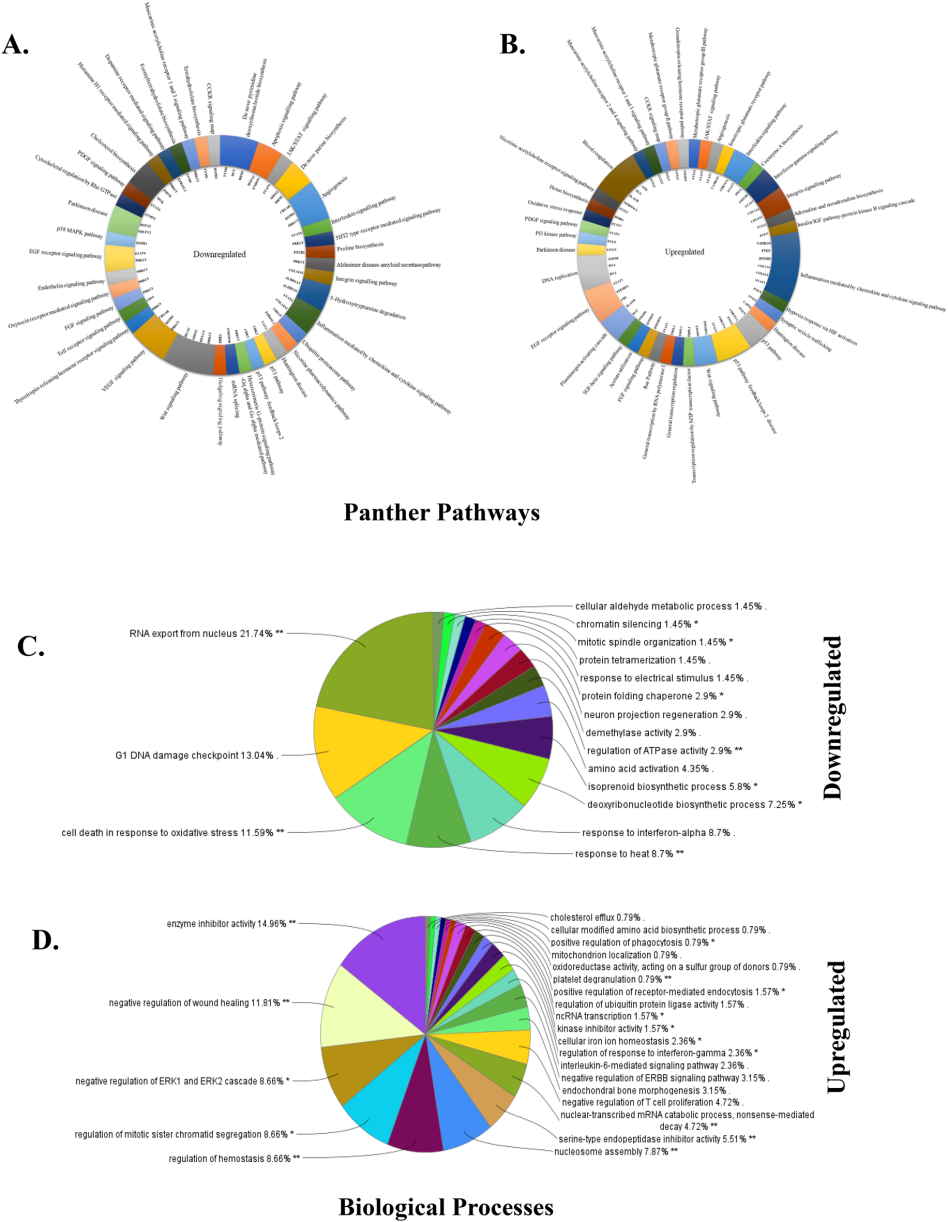
Bergenin causes the alteration in different pathways in cervical cancer cells. Representative distribution of (**A**) downregulated pathways with proteins involved in different ways using Cytoscape software. (**B**) Upregulated pathways with proteins involved in different ways using Cytoscape software. (**C**) Distribution of downregulated proteins in various biological networks using CLUEgo. (**D**) Distribution of upregulated proteins in various biological networks using CLUEgo.

A significant change was observed in the whole-cell proteome of the cells before and after treatment with bergenin. Based on the involvement in different pathways, all dysregulated proteins after bergenin treatment were categorized using a bioinformatic database PANTHER (protein analysis through evolutionary relationships) to establish a connection of downregulated (Fig. 3A) and upregulated (Fig. 3B) proteins with different pathways, biological processes, and molecular functions. In this analysis, we found that the prominent pathways downregulated by bergenin treatment are associated with inflammation, interleukin secretion, angiogenesis, proliferative p38 MAPK, EGFR signaling, JAK-STAT and histone H1 replication. Bergenin treatment also activated the p53 pathway, TGF beta, acetylcholine receptor, and oxidative stress response pathway, among others. Furthermore, the interrelations of downregulated (Fig. 3C) and upregulated (Fig. 3D) proteins in different biological networks were deciphered by using ClueGO (Cytoscape plug-in) analysis. This showed RNA export from nucleus (21.74%), G1 DNA damage checkpoint (13.04%), and cell death response to oxidative stress (11.59%) as top three downregulated cellular phenomena. In comparison, analysis of upregulated interaction indicated enzyme inhibitor activity (14.96%), negative regulation of wound healing (11.81%) and negative regulation of ERK1 and ERK2 signaling as the top three cellular phenomena.

### Bergenin inhibits migration and angiogenesis in cervical carcinoma cells 

**Figure 4:**
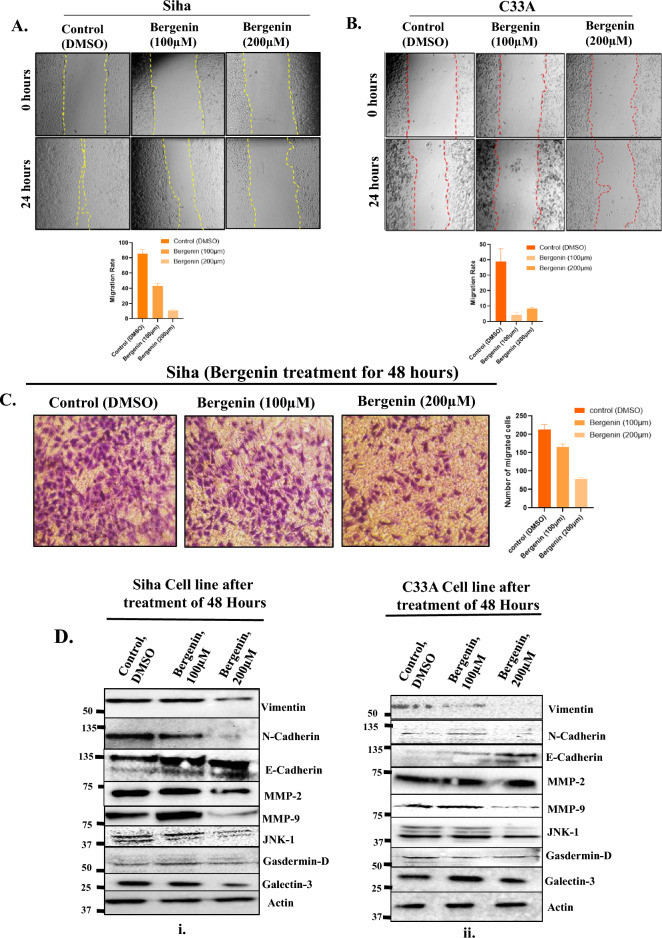
Bergenin influences migration and angiogenesis in cervical cancer cells. (**A**) Scratch assay was performed in SiHa cells with or without treatment of bergenin(100 µM and 200 µM) for 24 h. Pictures were taken at the start of the experiment (0 h) and 24 h later. Images of representative experiments are shown, and the means ± SD are illustrated as graphical representation. (**B**) Wound scratch assay was performed in C33A cells with or without treatment of bergenin (100 µM and 200 µM) for 24 h. Pictures were taken at the start of the experiment (0 h) and 24 h later. (**C**) Representative images from Transwell migration assay upon treatment of bergenin (100 μM and 200 μM) for 48 h in SiHa cells and means ± SD are illustrated as graphical representation. (**D**) Western blot of indicated markers of EMT and pathways after 100 μM and 200 μM bergenin treatment for 48 h in SiHa and C33A cell lines. The blots were cut before being subjected to hybridization with antibodies in the blotting procedure, and the original blots are provided in the supplementary information.

Our proteomic analysis revealed numerous candidate proteins implicated in angiogenesis, prompting us to conduct functional assays to investigate bergenin's impact on angiogenesis in cervical carcinoma cells. Metastasis is a complex multistep process characterized by the migration of cells from their point of origin and the emergence of disease-resistant clones. EMT and angiogenesis are critical mechanisms influencing metastasis and tumor progression^21^. To assess the effect of bergenin on EMT or migration, we performed the scratch assay on cervical cancer cells. The migration rate after bergenin (100 μM and 200 μM) treatment for 24 h was evaluated in SiHa (Fig. 4A) and C33A (Fig. 4B) cells. The gap closure percentage after bergenin treatment was significantly lower than that of the control group (DMSO treated) in both cell lines. These results revealed that bergenin inhibits the migration of cervical cancer cells. The results were further confirmed by transwell migration assay also revealed that the no. of migrated cells through the tarnswell were reduced upon treatment of bergenin (100 μM and 200 μM) for 48 h hrs in SiHa cells (Fig. 4C). To explore the effect of bergenin on migration and invasion, protein expression of EMT markers and metalloproteinases were evaluated in cervical cancer cell lines after bergenin (100 μM and 200 μM) treatment for 48 h by western blotting. Bergenin treatment increased the expression of E-cadherin (epithelial marker) and decreased expression levels of N-cadherin and Vimentin (mesenchymal markers) in both SiHa and C33A cells as indicated by western blot analysis (Fig. 4D). We also found a reduction in the level of MMP-9, a prominent metalloprotease that influences cellular migration (Fig. 4D). Galectin-3 is another protein that is required in many of these steps, like cell–cell adhesion and cell–matrix interactions^16^. A significant reduction in protein expression levels of Galectin-3 was observed in bergenin-treated cervical carcinoma cells (Fig. 4D). Overall, our data indicated that bergenin regulates migration and invasion of cervical cancer cells by modulating various factors involved in EMT and angiogenesis.

### Molecular docking and molecular dynamics simulation of galectin-3 and bergenin complex indicate lower binding affinity of bergenin to Galectin 3

**Figure 5:**
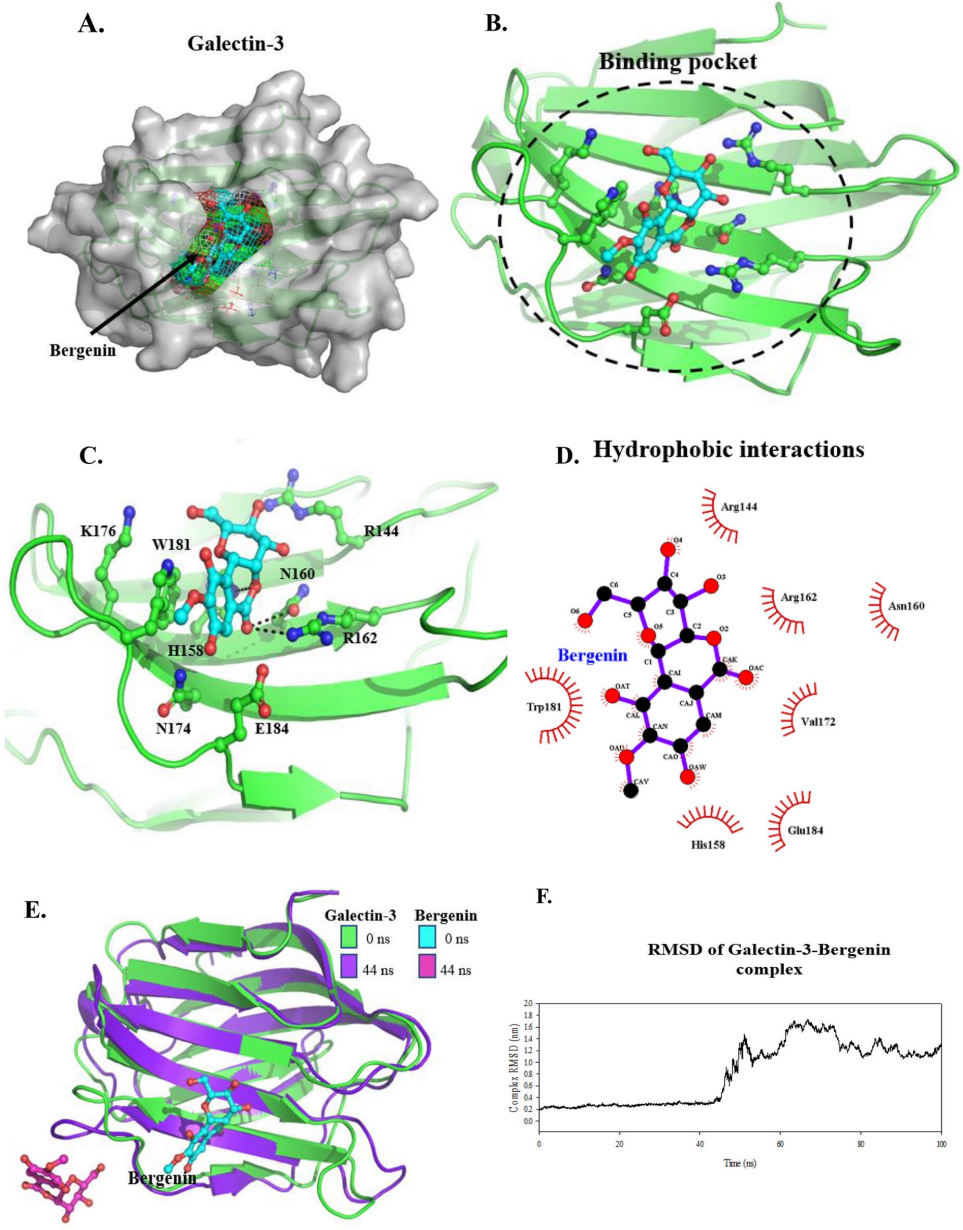
Molecular docking and molecular dynamic simulation of galectin-3 and bergenin complex. (**A**) The surface representation of galectin-3 and the docking/binding pocket of bergenin (ball-and-stick) are shown in the mesh. (**B**) Cartoon representation of bergenin binding site in galectin-3. (**C**) H-bonded interactions formed by docked bergenin compound with galectin-3. (**D**) The ligplot shows the hydrophobic and H-bonded interactions formed by bergenin. (**E**) Superimposition of galectin-3-bergenin complex at 0 and 100 ns of MD simulations. (**F**) RMSD plot of galectin-3-bergenin complex 100 ns MD simulations.

Since we saw a substantial reduction in the level of Galectin 3 in bergenin-treated cells, we did the molecular docking analysis to understand further the molecular basis of Galectins 3 and bergenin complex. The blind molecular docking showed that bergenin binds to Galectin-3 at the Carbohydrate recognition site of the protein with a binding energy of − 4.18 kJ/mol and a binding affinity of 857.13 µM (Fig. 5A,B)^16^*.* The best-docked conformer of bergenin forms two hydrogen-bonded interactions with residues Asn160 and Arg162 and a π–π interaction with Trp181 (Fig. 5C) and hydrophobic interactions with residues Arg144, His158, Asn160, Arg162, Val172, Glu182 and Trp181 (Fig. 5D). Surprisingly, during MD simulations, the bergenin molecule moved out of the binding site at 44 ns indicating that the compound has a lower binding affinity towards galectin-3 (Fig. 5E). The root-mean-square deviation (RMSD) clearly showed that the bergenin compound moved out of the binding site of the galectin-3-bergenin complex (Fig. 5F), indicating that there is a transient interaction between these molecules. The H-bond bond occupancy, the solvent-accessible surface area (SASA), the radius of gyration (Rg), and the root-mean-square fluctuation (RMSF) of the complex is shown in Figures S2A-2D.

### Molecular docking and molecular dynamics simulation of MMP-9 (Matrix Metalloprotease 9) and bergenin complex showed a strong binding affinity of Bergenin with MMP-9

**Figure 6:**
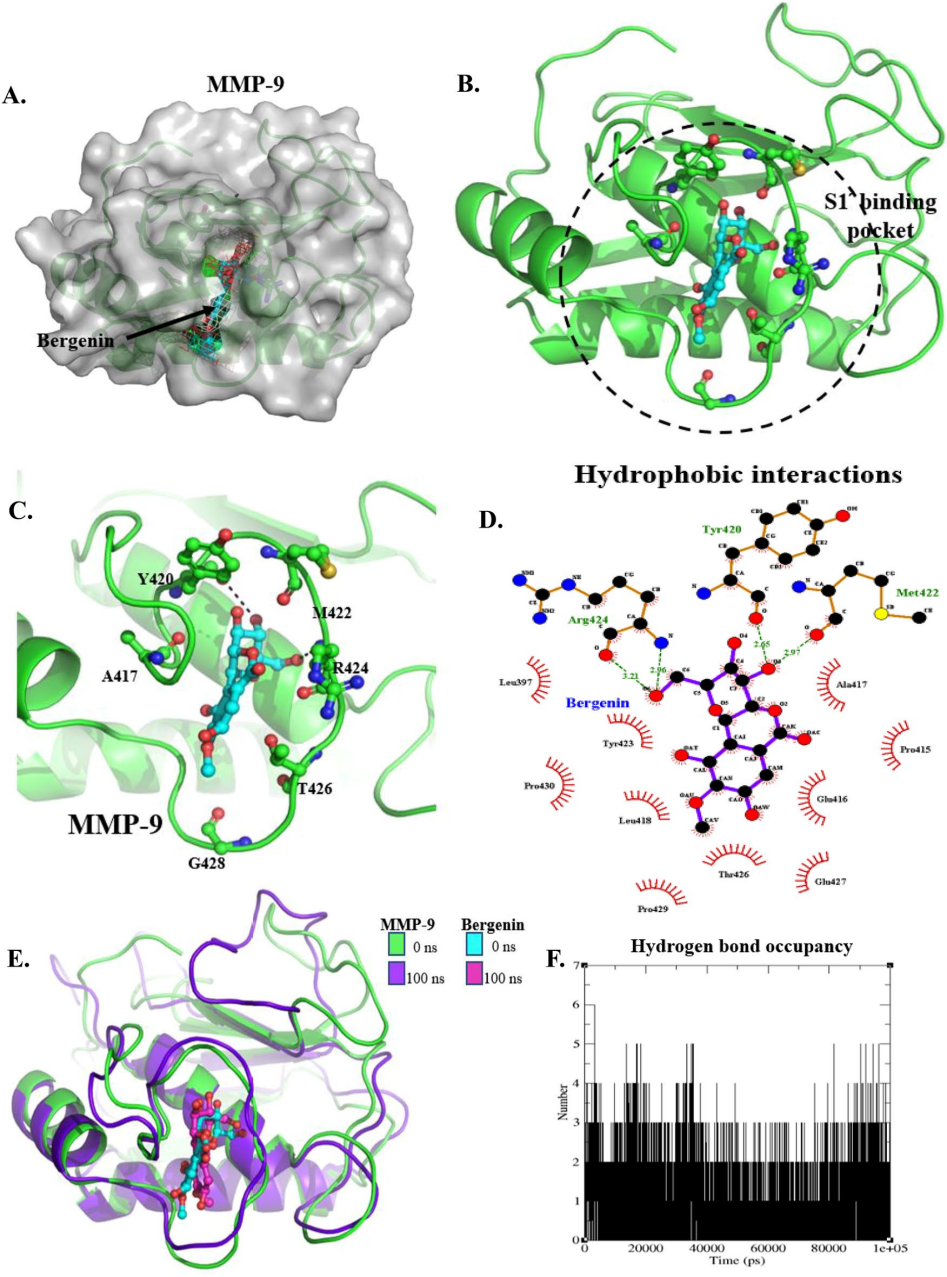
Molecular docking and molecular dynamic simulations of MMP-9 and bergenin complex. (**A**) The surface representation of MMP-9, along with the docking/binding pocket of bergenin (ball-and-stick) shown in the mesh. (**B**) Cartoon representation of bergenin binding site in MMP-9. (**C**) H-bonded interactions formed by docked bergenin compound with MMP-9. (**D**) The ligplot shows the hydrophobic and H-bonded interactions formed by bergenin (after docking). (**E**) Superimposition of MMP-9-bergenin complex at 0 and 100 ns of MD simulations. and (**F**) H-bond occupancy of the MMP-9-bergenin complex throughout 100 ns MD simulations.

We further carried out the molecular docking analysis of bergenin with MMP-9, another protein found to be significantly downregulated upon treatment by bergenin. The blind docking of bergenin with MMP-9 showed the compound binds at the S1′ pocket as described in the crystal structure complex of MMP-9 with a reverse hydroxamate inhibition^22–25^ (Fig. 6A). The bergenin forms two H-bonded interactions with the backbone atom of residues Tyr420 and Arg424 (Fig. 6C). It forms hydrophobic interactions with residues Leu397, Pro415, Glu416, Ala417, Leu418, Tyr423, Thr426, Glu427, Pro429, and Pro430 with a binding energy of − 8.39 kJ/mol and binding affinity of 702.38 nM (Fig. 6B–D). Upon analysis of 100 ns MD simulations of the MMP-9-bergenin complex, the compound was found to be stable inside the pocket, forming three H-bonds with Tyr420, Thr426, and Gly428 (Fig. 6E, Supplementary Fig. S3A) and 10 hydrophobic interactions with residues of MMP-9 (Supplementary Fig. S3A). The H-bond occupancy analysis of the 100 ns MD simulations suggested that the bergenin tends to form 6H-bonds (with an average of 2 H-bond) with MMP-9 (Fig. 6F). The RMSD, RMSF, SASA, and Rg also showed that the protein–ligand complex was stable throughout 100 ns MD simulations, indicating that bergenin has a higher affinity towards MMP-9 protein (Supplementary Fig. S3B–E). The MM/PBSA calculations also showed the compound bergenin stably interacts with MMP-9 and has a total binding of − 137.452 ± 13.327 kcal/mol with MMP-9 (Table 1) (Supplementary Fig. S3F,G). This is the first report showing that bergenin firstly reduced MMP-9 levels and secondly bergenin binds MMP-9 more efficiently as compared to galectin-3, suggesting a cumulative effect of bergenin on Galectin 3 and MMP 9 leads to reduced migratory characteristics in cervical carcinoma cells.

**Table 1:** Free energy calculation of Bergenin-MMP-9 complex by MMPBSA.

Type	Energy in kJ/mol
van der Waal energy	− 188.939 ± 11.172
Electrostatic energy	− 27.191 ± 7.329
Polar solvation energy	95.124 ± 12.939
SASA energy	− 16.446 ± 0.849
Binding energy	− 137.452 ± 13.327

## Discussion

Cervical cancer is one of the leading causes of cancer death among females worldwide. High-risk subtypes of the human papillomavirus (HPV) are associated with cervical cancer in the majority of cases. Although the incidence of cervical cancer has declined in recent years, especially in developing countries because of widespread vaccination and screening, cervical cancer still causes a serious threat to women’s reproductive health in developing countries where vaccine coverage is relatively low^26^. Many traditional natural compounds have recently gained widespread acceptance as additional or alternative treatments in modern medicine due to lower off-target effects^27^. Several novel anticancer compounds have been isolated from plants and have opened a new avenue for drug development against different cancers. These natural compounds provide an alternative approach to the limitations of conventional chemotherapy (severe side effects and inefficacy due to the occurrence of multi-drug resistance)^28^. In the current study, we explored the effect of bergenin, an important plant-derived compound on the cervical cancer cells as its anticancer properties have recently been studied in very few cancers^13,29^. In the current study, we performed whole-cell label-free proteome analysis to identify the potential targets of bergenin in cervical cancer.

First we examined the effect of bergenin on the cell viability of cervical cancer cells. The results suggested that the bergenin treatment reduced cell viability of cervical cancer cells (SiHa and C33A), irrespective of their HPV status. Additionally, typical cellular apoptotic morphological alterations were seen in both cell lines, including cell shrinkage, and fragmentation into membrane-bound apoptotic bodies. To understand the mechanism of action of bergenin in cervical cancer and its effects on different pathways, we did a whole-cell proteome profile (label-free protein quantification) of SiHa cervical cancer cells treated with bergenin. The results of label-free protein quantification suggest that bergenin induces the dysregulation of a different set of proteins. Panther server categorized all the dysregulated proteins based on their cellular localization, gene ontologies, pathways, and protein class. Bergenin was found to downregulate several proteins in diverse cellular pathways like Wnt (PRKC1, HLTF), VEGF (CRYAB, HSPB1), JAK-STAT(STAT-6), Apoptosis (HSPA2, ENDOG), angiogenesis (CRYAB, PRKC1), p53 pathway (CDK2), and inflammation mediated by chemokine and cytokine signaling (STAT-6, COL14A1). The upregulated pathways PI3 kinase pathway, inflammation mediated by chemokine and cytokine signalling pathway (IFNGR1, COL1A1, COL6A1), CCKR signalling (PTEN), and STAT-1 and many more pathways regulating cell growth and angiogenesis. In our analysis, it was seen that most proteins influenced following bergenin treatment had tumor suppressive role. Further involvement of dysregulated and unique proteins in different biological processes was analyzed using Cluego plugin which indicated that many of these pathways are intricately connected and their relation needs to be explored further in future studies.

Interestingly, we saw that many factors associated with angiogenesis were also downregulated after bergenin treatment, thus necessitating to explore the effect on protein markers of angiogenesis and metastasis. Metastasis requires the involvement of a wide array of cellular mechanisms led by cytoskeleton dynamics and molecular alterations such as expression of adhesion and proteolytic enzymes^30^. EMT and angiogenesis are essential mechanisms influencing metastasis and tumor progression^21^. EMT is the physiological or pathological conversion of epithelial cells to mesenchymal cells, in which cell adhesion and polarity are lost, and invasive properties are acquired^31^. The ECM is a complex network composed of extracellular macromolecules such as collagen and glycoproteins. The ECM restricts the migration of normal cells, while tumor cells and macrophages secrete matrix metalloproteinases such as MMPs. MMP2 and MMP9 degrade ECM to promote cancer cell invasion^32^. A recent study showed that bergenin has potential anticancer properties in bladder cancer cells by targeting multiple hallmarks of cancer. According to this study, bergenin treatment reduced the levels of N-cadherin and vimentin expression, which are indicators of the mesenchymal phenotype, and suggests that bergenin potentially regulates EMT in bladder cancer^14^. Another study identifies bergenin as a pharmacologically significant compound in *Excoecaria agallocha* (L.) leaf extract, which exhibits anti-tumor activities and inhibits the proliferation of cervical cancer cells (SiHa)^15^. However, this study did not explore the molecular pathways modulated by bergenin. The data in the current study have underlined exact targets modulated by bergenin. We show that bergenin inhibits cervical cancer cells' migration and invasion via modulating several factors involved in angiogenesis and EMT. The migration rate following bergenin treatment was assessed in both cervical cancer cell lines (SiHa and C33A) using a scratch wound assay which displayed a significant reduction in migratory phenotype following bergenin treatment. Additionally, increased levels of E-cadherin (epithelial marker) and reduced N-cadherin and Vimentin (mesenchymal marker) were observed in bergenin-treated cervical cancer cells. There was also a reduction in MMP-9 and galectin-3 protein expression levels following bergenin treatment. MMP-9 and Galectin-3 are important proteins which regulate cell–cell adhesion and interactions with the cell matrix, as well as angiogenesis and metastasis in cancer. A previous study describes the bergenin as a potential inhibitor of galectin-3^16^. Based on the inhibitory effect of bergenin on galectin-3 and MMP-9, we performed the molecular docking using AutoDock4.2.6^33^ and further MD simulations studies using GROMACS-5.0.7. to understand the binding affinity and molecular interactions of the ergenin with Galectin-3 and MMP-9.We found that bergenin binds to Galectin-3 at the protein's carbohydrate recognition site with a binding energy of − 4.18 kJ/mol and a binding affinity of 857.13 M, but the bergenin molecule unexpectedly left the binding site during MD simulations at 44 ns, showing that the drug had a lower affinity for galectin-3. However, the crystal structure complex of MMP-9 with a reverse hydroxamate inhibitor interacts at the S1 pocket, and bergenin forms two H-bonded contacts with the backbone residues Tyr420 and Arg424, and hydrophobic interactions with residues Leu397, Pro415, Glu416, Ala417, Leu418, Tyr423, Thr426, Glu427, Pro429, and Pro430. The MMP-9-bergenin complex was examined using 100 ns MD simulations, and it was discovered that bergenin was stable inside the pocket and had a greater affinity for the MMP-9 protein. Bergenin has a stable interaction with MMP-9 as compared to galectin-3. This study for the first time provides deep insight into bergenin-mediated toxicity in cervical carcinoma cells and identifies and validates the potential targets of bergenin, especially Galectin 3 and MMP-9 along with proposing many new ones. This study offers vital insights into the molecular mechanisms underlying bergenin-induced cytotoxicity in cervical carcinoma cells, highlighting the need for further investigations in alternative models such as murine models. These models allow for a more comprehensive assessment, particularly regarding concerns like bioavailability that often arise with natural products. The potential development of bergenin in novel nanoformulations aimed at enhancing its bioavailability at tumor sites presents a promising avenue to transition this significant natural product from experimental settings to clinical application.This study also leaves many open-ended questions regarding validation of targets that are involved in controlling other hallmarks of cancer which will be the subject of our future studies.

## Materials and methods

### Reagents

Bergenin was purchased from MedChemExpress USA (Cat. No.: HY-N0017) and disolved in dimethyl sulfoxide (DMSO) for preparing the stock solution (10 mM) and diluted in different doses before application. For cell culture, we used fetal bovine serum (FBS; 10270106, ThermoFisher) and DMEM medium (11995-065, Gibco), 0.25% Trypsin–EDTA (25200-056, Gibco) and DMSO (28580, SRL). For molecular assays, bis-acrylamide (38862, SRL), Tris (252859, Sigma), ammonium persulfate (431532, Sigma), and sodium dodecyl sulfate (11667289001, Sigma). Halt™ Protease Inhibitor Cocktail (100×) (78430, Thermo), thiazolyl blue tetrazolium bromide (MTT; M5655), Pierce™ BCA Protein Assay Kit (23225, ThermoFisher, MA, USA) were obtained from source as indicated. Antibodies like vimentin (A19607), E-cadherin (A20798), N-cadherin (A19083), MMP-2(A19080), MMP-9 (A0289), JNK1 (A0288), Gasdermin (A20728), galectin 3 (A11198) were purchased from Abclonal technology MA, United States while beta-actin (sc-47778) was purchased from Santa cruz Technology USA.

### Cell lines

HPV-positive SiHa and HPV-negative C33A human cervical cancer cell lines were procured directly from the NCCS (National centre for cell sciences), Pune, India. All cell lines were grown in DMEM (Gibco, USA) supplemented with 10% FBS, 5 U/ml penicillin, 0.5 U/ml streptomycin, and 2 mM glutamine.

### MTT assay

Cell viability was measured by 3-(4,5-dimethylthiazol-2-yl)-2,5-diphenyltetra-zolium bromide (MTT) assay. SiHa and C33A cells were trypsinized and resuspended in a DMEM medium. Then Cells were plated at a concentration of 5 × 10^3^ cells/well in 96-well plates (NEST technology, CA, USA) with 100 μl DMEM medium and grown for 24 h. Cells were treated with serial dilutions of bergenin concentrations range (0–400 µM) using appropriate controls for 24 and 48 h and then washed with phosphate-buffered saline. After treatment, 10 µl of 5 mg/ml MTT in PBS was added to each well and incubated at 37 °C for 4 h. After that, 100 μl of dimethyl sulfoxide was added to each well at 37 °C and the optical absorbance at 570 nm was determined using a microplate reader (BioTek, VT, USA).

### Annexin V-PE/7-AAD staining assay

SiHa cervical carcinoma Cells were treated with bergenin(100 µM and 200 µM) for 48 h. The cells were washed twice with cold PBS then resuspended with 1× Binding buffer at a concentration of 1 × 10^6^ cells/ml and then cells were stained with Annexin-V-APC (5 µl) (BD Biosciences) and 7-AAD (5 µl) (BD Biosciences),Catalogue No. 550474 in dark for 15 min at room temperature. After incubation a minimum of 20,000 cells were acquired using Gallios flow cytometer (Beckmann coulter) and the data was analysed using Kaluza software (Beckmann coulter). Cells stained with 7AAD can be used as a control to set up compensation and quadrants for Annexin-V. Therefore, fluorescence minus one (FMO) for Annexin-V (contains only 7AAD) has been used to set a gating for positively stained cells.

### Wound healing/scratch assay

Cervical cancer cell lines C33A and SiHa were grown in complete culture medium (DMEM with 10% FBS) until they reached full confluence. A wound was generated by gently creating an incision using a 200 µl micropipette tip, followed by a PBS wash to remove cellular debris. Subsequently, the PBS was replaced with fresh culture medium containing bergenin at a concentration of 100 µM and 200 µM, and the cells were then incubated at 37 °C for 24 h. A control group was treated with media containing DMSO. Following incubation, the cells were washed twice with PBS, and the wound was visualized using a TS100 Eclipse microscope from Nikon. Data analysis was performed using ImageJ software,

### Transwell migration assay

The Transwell migration assay utilized 24-well inserts with polyethylene terephthalate membranes (8.0 μm pore size, Corning). In this migration assay, 5 × 10^4^ cells were suspended in 200 μl of 1% FBS DMEM medium containing bergenin at concentrations of 100 µM and 200 µM, while DMSO was used as the control. These cell suspensions were loaded into the upper chamber of the Transwell. Subsequently, 600 μl of DMEM medium containing 10% FBS was added to the lower well to serve as a source of chemo-attractants. After a 24-h incubation period, the cells on the lower surface of the insert were fixed using methanol and stained with 0.5% crystal violet. Stained cells were visualized and captured using a TS100 Eclipse microscope (Nikon). Cell counting was performed by randomly selecting three fields, and this experiment was repeated three times. The results are presented as means ± SD.

### Western blotting

Cervical cancer cells SiHa and C33A were treated with bergenin (100 µM and 200 µM) for 48 h and lysed by RIPA Lysis buffer to obtain total protein. Protein concentration was assessed by using a BCA protein assay kit. All cell lysates containing an equal amount of protein were suspended in 4× Lammeli buffer, boiled for 5 min, separated on sodium dodecyl sulfate–polyacrylamide gel electrophoresis (10–15%), and transferred to the PVDF membranes. The membranes were blocked using 5% BSA/Non-fat Dry milk and then incubated with primary antibodies overnight, washed and incubated with secondary antibodies, the next day followed by washing. The blots were cut before being subjected to hybridization with antibodies in the blotting procedure, and the original blots of each blots are provided in the supplementary information. Then, the blots were visualized using enhanced chemiluminescence by a gel imaging system.

### Sample preparation for whole cell label-free quantification (LFQ)

The SiHa cell pellets (both untreated and bergenin treated) were washed with 1× Tris saline Buffer Saline (TBS) and then treated with the protease inhibitor (Roche). Additionally, the GN buffer (6 M guanidium HCl and 0.1 M Tris pH 8.5) was added to the cell pellets, followed by resuspension and vertexing. Then, the samples were kept on ice and subjected to 3 min of sonication (1 s pulse and 1 s rest) for nucleic acid breakdown and membrane protein solubilization. The samples were centrifuged for 20 min at 10,000 rpm for complete lysis. The supernatant was collected into a fresh tube, followed by the quantification using the Bradford method (so that an equal amount of whole protein from different samples can be used for in-digestion protocol). 25 μl of SiHa cell lysates were reduced with 5 mM TCEP and further subjected to 50 mM iodoacetamide for alkylation followed by Trypsin digestion (1:50, Trypsin/lysate ratio) at 37 °C for 16 h. Then, the C18 silica cartridge was used to remove the salts from the whole cell SiHa lysate digests, followed by freeze drying using the speed vac. The dried whole cell lysate digested pellets were resuspended in Buffer A (5% acetonitrile, 0.1% formic acid).

### Mass spectrometric analysis of peptide mixtures and data processing

For mass spectrometric analysis, the same methodology as described previously was used^34^. The Thermo Fisher Scientific EASY-nLC 1000 system coupled to QExactive (Thermo Fisher Scientific) is equipped with the nano electrospray ion source. A total of 1 μg of peptide mixture concentration (for both Bergenin untreated and treated samples) was resolved via a PicoFrit column (15 cm, 10 μm tip, 360 μm outer diameter, 75 μm inner diameter) filled with C-18 resin (2 μm) (Dr. Maeisch, Germany). The peptides were further loaded with Buffer A and then eluted using 0–40% of buffer B in gradient (95% acetonitrile, 0.1% formic acid) at 300 nL/min of flow rate for 100 min. Finally, a data-dependent top 10 method was used for acquiring the MS data by dynamically choosing the most abundant precursor ions from the survey scan.

The experiment was done in three replicates and the significant proteins (by t-test) showing the abundance ratio (fold change compared to control) of ≥ 1.5 (both up and downregulation) were used for further processing. For analysis, two categories were made; the first category includes the downregulated proteins and the proteins unique to untreated cells, whereas the second category consists of upregulated proteins and the proteins unique to the bergenin-treated cells. The supplementary table, denoted as Supplementary Table S1, contains a comprehensive list of proteins that were dysregulated and unique both before and after bergenin treatment.The panther server was used for making panther pathways for both downregulated and upregulated proteins separately. Also, the cellular component (or compartmentalization) of the dysregulated proteins was created using the panther server. The biological processes of dysregulated and unique proteins were found by the Cluego plugin using Cytoscape^34–36^.

### Molecular docking and MD simulations

To understand the binding affinity and molecular interactions of the bergenin with Galectin-3 and MMP-9, the molecular docking study was performed using AutoDock4.2.6^33^. The crystal structures of Galectin-3 (PDB ID: 1KJL) and MMP-9 (PDB ID: 2OW)^22,37^ were used for docking studies, and the coordinates of bergenin were obtained from PubChem. Blind docking was performed for both proteins without defining the binding site. As described in previous studies, the docking was carried out using the Lamarckian Genetic Algorithm with default docking parameters^38–40^. In both docking studies, ten best-docked poses of bergenin were compared, and the lowest binding energy conformation in the major cluster was selected for further 100 ns MD simulations studies using GROMACS-5.0.7^41,42^ with GROMOS 54a7 force field^43^. Each protein-bergenin complex was placed in a 10 Å cubic box solvated with water model SPC216 and equilibrated at 300 K^44^. The NVT and NPT calculations were performed for both complexes, followed by a 100 ns production. The detailed protocol used is similar to the study by Malhotra et al.^40^. The Root mean square deviation (RMSD), Root mean square fluctuation (RMSF), Solvent accessible surface area (SASA), and Radius of gyration (Rg) were calculated using GROMACS-5.0.7^42^. The H-bond occupancy throughout the MD simulations was also calculated for the stable complex. The MM/PBSA calculations were performed to determine the free energy of the complex using the g_mmpbsa module by GROMACS-5.0.7 using the default parameters^45,46^.

### Statistical analysis

Unless otherwise stated, data are expressed as means ± SEM. Comparisons of three or more sets of data were conducted by one-way ANOVA or two-way ANOVA. All statistical analyses were conducted with GraphPad Prism 8 software (GraphPad Software, San Francisco, CA). Differences were considered statistically significant at p < 0.05.

### Supplementary Information

Supplementary Figures.

Supplementary Tables.

## Data Availability

The dataset created for the study titled "Bergenin inhibits growth of human cervical cancer cells by decreasing Galectin-3 and MMP-9 expression" has been successfully submitted to ProteomeXchange through the PRIDE database. The mass spectrometry proteomics data are now available in the ProteomeXchange Consortium via the PRIDE partner repository, and can be accessed using the dataset identifier PXD043359. For further details regarding the submission, the project is named "Bergenin inhibits growth of human cervical cancer cells by decreasing Galectin-3 and MMP-9 expression," with the project accession PXD043359, and the project DOI is not applicable and reference: 1-20230628-050348-2745934.
